# Improving reporting of infant deaths, maternal deaths and stillbirths in Haryana, India

**DOI:** 10.2471/BLT.15.157693

**Published:** 2016-05-02

**Authors:** Preeti H Negandhi, Sutapa B Neogi, Sapna Chopra, Amit Phogat, Rupinder Sahota, Ravikant Gupta, Rakesh Gupta, Sanjay Zodpey

**Affiliations:** a Indian Institute of Public Health Delhi, Public Health Foundation of India, Plot 47, Sector 44, Institutional Area, Gurgaon – 122002, Haryana, India.; b National Health Mission, State Government of Haryana, Panchkula, India.; c State Government of Haryana, Chandigarh, India.

## Abstract

Underreporting hampers the accurate estimation of the numbers of infant and maternal deaths and stillbirths in India. In Haryana state, a surveillance-based model – the Maternal Infant Death Review System – was launched in 2013 to try to resolve this issue. The system is a mixture of routine passive data collection and active surveillance by specially recruited and trained field volunteers. The volunteers gather the relevant data from child day-care centres, community health centres, cremation grounds, hospitals, the municipal corporation’s offices and primary health centres and regularly visit health subcentres. The collected data are triangulated against the standard death registers and discussions with relevant community members. The details of any unregistered death are rapidly uploaded on the system’s web-based platform. In April 2014, we made field observations, reviewed records and conducted in-depth interviews with the key stakeholders to see if the system’s performance matched the state government’s planned objectives. The data collected indicate that implementation of the system has led to quantitative and qualitative improvements in reporting of infant and maternal deaths and stillbirths. Completeness and consistency in the reporting of deaths are essential for focused policy and programmatic interventions and there remains scope for improvement in Haryana via further reform and changes in policy. The model in its current form is potentially sustainable and scalable in similar settings elsewhere.

## Résumé

En Inde, le sous-signalement empêche d'estimer correctement le nombre de décès infantiles et maternels et de mortinaissances. Dans l'État d'Haryana, un modèle basé sur la surveillance – le *Maternal Infant Death Review System* – a été lancé en 2013 afin de tenter de résoudre ce problème. Ce système mélange recueil passif de données de routine et surveillance active par des bénévoles de terrain spécialement recrutés et formés à cet effet. Ces derniers recueillent des données auprès de garderies, de centres de santé communautaires, de crématoriums, d'hôpitaux, de centres de soins primaires et des bureaux des municipalités et se rendent régulièrement dans des centres de soins secondaires. Les données ainsi recueillies sont triangulées par rapport aux registres standards des décès et aux discussions avec des membres de la communauté. Les détails de tout décès non enregistré sont rapidement chargés sur la plate-forme Internet du système. En avril 2014, nous avons effectué des observations de terrain, examiné des registres et mené des entretiens approfondis avec les principales parties prenantes afin de voir si les performances du système répondaient aux objectifs du gouvernement de l'État. Les données recueillies indiquent que la mise en œuvre de ce système a entraîné une amélioration quantitative et qualitative du signalement des décès infantiles et maternels ainsi que des mortinaissances. L'exhaustivité et la cohérence du signalement des décès sont essentielles pour avoir des politiques ciblées et des interventions programmatiques, et l'État d'Haryana présente d’autres possibilités d'amélioration, via d'autres réformes et des changements stratégiques. Le modèle, dans sa forme actuelle, est potentiellement utilisable à long terme et transposable dans des lieux similaires.

## Resumen

La escasez de informes obstaculiza una estimación exacta de las cifras de muertes maternas e infantiles y mortinatos en India. En 2013, en el estado de Haryana, se lanzó un modelo basado en el seguimiento (el Sistema de Análisis de la Mortalidad Infantil y Materna) para tratar de resolver este problema. El sistema combina una recopilación de datos rutinarios pasivos y un seguimiento activo realizados por voluntarios contratados capacitados en este campo. Los voluntarios reúnen información relevante de guarderías, centros de salud, terrenos destinados a incineraciones, hospitales, oficinas de la corporación municipal y centros de atención primaria, y visitan con asiduidad subcentros de salud. Los datos recopilados se triangulan según los registros normalizados de fallecimientos y análisis con miembros relevantes de la comunidad. Los detalles sobre todas las muertes no registradas se introducen con rapidez en la plataforma en línea del sistema. En abril de 2014, se realizaron observaciones de campo, se analizaron los registros y se llevaron a cabo entrevistas en profundidad con las partes interesadas fundamentales para comprobar que el rendimiento del sistema se equiparaba con los objetivos planificados por el gobierno estatal. Los datos recopilados indican que la implementación del sistema logró mejoras cuantitativas y cualitativas a la hora de redactar informes sobre la mortalidad infantil y materna y los mortinatos. Es fundamental que los informes sobre los fallecimientos sean minuciosos y coherentes para poder realizar intervenciones políticas y programáticas, y sigue existiendo margen para implementar mejoras en Haryana mediante más reformas y cambios de las políticas. El modelo actual puede mantenerse y ampliarse en otras ubicaciones similares.

## ملخص

يؤدي التقصير في تقديم البلاغات إلى الانتقاص من التقديرات الدقيقة لأعداد الوفيات بين الرضع والأمهات ووفيات الأجنة في الهند. في ولاية "هاراينا" تم استقدام نظام لمراجعة حالات وفيات الأمهات والرضع – وهو عبارة عن نموذج يقوم على أساس استقصائي – في عام 2013 لمحاولة حل هذه المشكلة. ويمثل النظام خليطًا من الأساليب الروتينية لجمع البيانات بطرق سلبية والاستقصاء النشط من جانب متطوعين ميدانيين تم انتدابهم وتدريبهم بشكل خاص. يتولى المتطوعون جمع البيانات ذات الصلة من مراكز الرعاية النهارية للأطفال، ومراكز الصحة المجتمعية، وساحات حرق جثث المتوفين، والمستشفيات، ومكاتب البلدية، ومراكز الصحة الأساسية، كما يتولون زيارة المراكز الصحية الفرعية بشكل منتظم. وتم تقسيم البيانات التي تم تجميعها إلى قطاعات مثلثة بناءً على السجلات المعيارية للوفيات والمناقشات التي جرت مع أفراد المجتمع المعنيين. ويتم تحميل تفاصيل أي حالة وفاة غير مسجلة بشكل سريع عبر واجهة الإنترنت الخاصة بالنظام. وفي شهر أبريل/نيسان 2014، قمنا بإجراء ملاحظات ميدانية، ومراجعة السجلات، وإجراء مقابلات متعمقة مع الجهات المعنية الأساسية للوقوف على ما إذا كان أداء النظام يطابق الأهداف المقصودة لحكومة الولاية. وقد أشارت البيانات التي تم جمعها إلى أن تطبيق النظام قد أدى إلى حدوث تحسينات نوعية وكمية في أنشطة الإبلاغ عن وفيات الرضع والأمهات والأجنة. من الضروري الإبلاغ عن الوفيات بشكل كامل وثابت المستوى وذلك لتخطيط عمليات التدخل المستندة إلى اتباع سياسات مُركّزة وبرامج منهجية، ولا يزال هناك مجال للتحسن في "هاريانا" من خلال إجراء المزيد من الإصلاحات والتغييرات في السياسات. ويفترض في هذا النموذج بشكله الحالي أن يتوفر له عنصر الاستدامة وقابلية التطوير في بيئات مشابهة بأماكن أخرى.

## 摘要

在印度，漏报现象阻碍了对其婴儿和孕产妇死亡数字的准确估计。 哈里亚纳邦于 2013 年推出了基于监督的模式——母婴死亡审查系统，以试图解决该问题。 该系统采用常规被动数据收集和主动监督相结合的方式。具体操作由经过专门招聘和培训的现场志愿者实施。 志愿者们从幼儿日托中心、社区卫生中心、火葬场、医院、市政公司办事处和初级保健中心收集相关数据并且定期访问保健分中心。 根据标准死亡登记以及与相关社区成员的讨论，对所收集的数据进行三角测量。 任何未经登记的死亡详情立即被上传至基于网络的系统平台。 2014 年 4 月，我们与主要利益相关者进行了现场观察、审查了记录同时开展了深入的访问，以查明该系统的性能与邦政府规划的目标是否匹配。 收集的数据表明该系统的实施在定量和定性方面改善了婴儿、孕产妇死亡率以及死产的报告。 死亡报告的完整性和一致性对于制定有的放矢的政策和开展有计划的干预至关重要。哈里亚纳邦在政策的深入改革和变更方面仍然有待提高。 当前形式的模式在其他背景相似的地方具有潜在的可持续性和可扩展性。

## Резюме

Неполное освещение ситуации препятствует точному определению числа младенческих и материнских смертей и мертворождений в Индии. Для решения этой проблемы в штате Харьяна в 2013 году была запущена модель на основе обзорного наблюдения — система обзора материнских и младенческих смертей. Эта система сочетает в себе периодический пассивный сбор данных и активное наблюдение, осуществляемое специально набранными и подготовленными выездными группами добровольцев. Добровольцы осуществляют сбор данных в детских садах, центрах охраны здоровья, местах сожжения трупов, больницах, офисах местной администрации и центрах первичной медико-санитарной помощи и регулярно посещают вспомогательные медицинские центры. Собранные данные интерпретируются методом триангуляции путем их сопоставления с данными стандартных реестров смертей и обсуждения с членами соответствующих общин. Сведения обо всех незарегистрированных смертях оперативно загружаются на платформу системы, основанную на веб-сайте. В апреле 2014 года были осуществлены полевые наблюдения, проанализированы записи и проведены содержательные собеседования с основными заинтересованными лицами, чтобы определить, позволяют ли возможности системы достичь целей, установленных правительством штата. Собранные данные свидетельствуют о том, что внедрение системы привело к количественным и качественным улучшениям сообщаемой информации по младенческим и материнским смертям и мертворождениям. Полнота и непротиворечивость отчетности по смертям крайне необходимы для формирования целенаправленной политики и осуществления программных мер, и в штате Харьяна по-прежнему существуют возможности для улучшения ситуации путем дальнейшего реформирования и изменений в политике. Данная модель в ее текущей форме является потенциально устойчивой и может быть применена повсеместно в любых других масштабах при наличии сходных условий.

## Introduction

Each year in India, there are approximately 28 million pregnancies, 26 million live births, 67 000 maternal deaths and a million neonatal deaths.^1^ There are about 42 infant deaths per 1000 live births,^2^ five stillbirths per 1000 deliveries^3^ and 178 maternal deaths per 100 000 live births.^4^ The National Health Mission, which was launched in 2005, marked a turning point in the history of India’s health-care system. Although the mission had many objectives, some of its main goals were reductions in infant and maternal mortality as well as a general improvement in the quality of health-care services via a sustainable system.

The northern state of Haryana is India’s second wealthiest state, in terms of its annual per capita income – about 2139 United States dollars in 2012–2013.^5^ Despite this relative prosperity, Haryana records rates of infant mortality – 42 infant deaths per 1000 live births^2^ – and stillbirth – nine stillbirths per 1000 births^3^ – and a maternal mortality ratio – 146 maternal deaths per 100 000 live births^4^ – that are similar or worse than the national mean values. It is, however, unlikely that the official national and state records of infant and maternal mortality and stillbirth are accurate. Although such records may be supplemented by data collected by routine health services or in sample surveys, they are usually largely based on vital statistics.^6^ In most low- and middle-income countries, the incomplete registration of births and deaths results in inaccurate vital statistics.^7^ In India, the registration of births has recently improved but the registration of deaths remains generally poor – although there have been interventions to improve the reporting of maternal deaths in the states of Kerala, Punjab, Tamil Nadu and West Bengal.^8^

Surveillance can help in the control and prevention of diseases of public health importance by facilitating planning, evaluation and the formulation of research hypotheses.^9^ In Haryana, a surveillance-based model – the Maternal Infant Death Review System – was launched in 2013 to try to improve state-wide estimates of infant and maternal mortality and stillbirth rates. Below, we describe the review system and the results of an evaluation of its impact that we conducted, according to the relevant guidelines of the United States Centers for Disease Control and Prevention.^9^

## The review system

### Genesis

Although the Indian health management information system is intended to provide facility-level reports on maternal and infant deaths, is not yet very robust and the information it provides remains incomplete.^10^ In 2012, the National Health Mission in Haryana therefore designed a centralized system of passive surveillance to gather better information on maternal deaths, infant deaths and stillbirths from facilities at various levels of the health-care system. In April 2013, the addition of active surveillance to this system was piloted in Karnal district. After a brief evaluation indicated that three deaths were being reported in Karnal for every one being detected by passive surveillance (V Chayal, Post Graduate Institute of Medical Sciences, Rohtak, India, personal communication, 2015), the combination of passive and active surveillance – i.e. the Maternal Infant Death Review System – was gradually rolled out across the state. From September 2013, the whole of Haryana was covered by the review system.

### Description

**Fig. 1:**
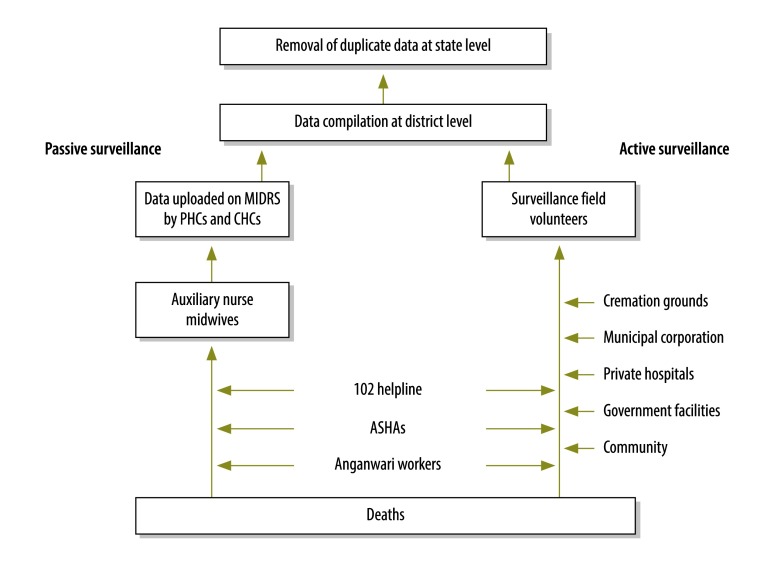
Flow of data in the active and passive surveillance of maternal and infant deaths in Haryana, India, 2013

ASHAs: accredited social health activists; CHCs: community health centres; MIDRS: Maternal Infant Death Review System; PHCs: primary health centres.

Note: The 102 helpline is a free referral transport system for pregnant women and infants.

The review system combines passive surveillance on infant and maternal deaths and stillbirths with active surveillance of the same events (Fig. 1). For the passive surveillance, frontline health-care workers and medical officers are asked to report, to their district-level authorities, every abortion, delivery, infant death, pregnancy, maternal death, stillbirth and vaccination and all antenatal and postnatal care and contraception provided in their area and community. The district-level managers are then asked to upload this information daily on the review system’s web-based platform. The standard case definitions of the World Health Organization (WHO) are used to categorize the deaths as infant, maternal or stillbirth.^11^^–^^13^

The active surveillance component of the review system is managed by 13 trained surveillance field volunteers, who were specially recruited and trained. These volunteers are asked to visit all the higher level facilities – i.e. the child day-care centres known as anganwadi centres, community health centres, cremation grounds, hospitals, the municipal corporation’s offices and primary health centres. They retrieve information from registers and discussion with staff, on any unreported maternal deaths, infant deaths and stillbirths. Having collected any relevant data, the volunteers must then visit the relevant lower level facilities, such as health subcentres, to verify the data with frontline health workers and check antenatal-care and postnatal-care registers. During verification, if the volunteer confirms the discovery of an infant or maternal death or stillbirth that has not been reported so far, that event is recorded on the web-based platform. In discussions with the relevant frontline health workers, the volunteers investigate why each previously unreported death or stillbirth had not been reported and educate the workers on the importance of reporting all deaths and stillbirths.

All women who die within nine months of registering for antenatal care or within two months of registering for postnatal care are classified as maternal deaths by the volunteers.

The volunteers are also encouraged to interact with community members and to visit households in the village or urban area where a death of interest has taken place, to verify the cause of death whenever there is any doubt.

Although some of the state’s private hospitals initially refused to share information on in-hospital deaths and stillbirths, all subsequently agreed to share such information with the state government and volunteers provided the hospitals’ names were not disclosed. The hospitals’ managers were assured that no legal action would be taken against them as a result of the information they shared.

As a single death may be reported to the review system more than once, for instance by different health workers at district and state levels, the review system’s platform is designed to highlight and remove duplicate data.

### Evaluation

In April 2014, we made field observations, reviewed records and conducted in-depth interviews with the key stakeholders and the volunteers to see if the review system’s performance matched the state government’s planned objectives. We held several rounds of formal and informal discussions to investigate the process, strengths and challenges of implementing the review system and the review system’s effectiveness and robustness. We investigated how each death registered by the review system had come to be reported and compared how the perceived trends in infant and maternal mortality in Haryana between 2012 and 2014 differed according to the source of the primary data.

### Effectiveness

**Fig. 2:**
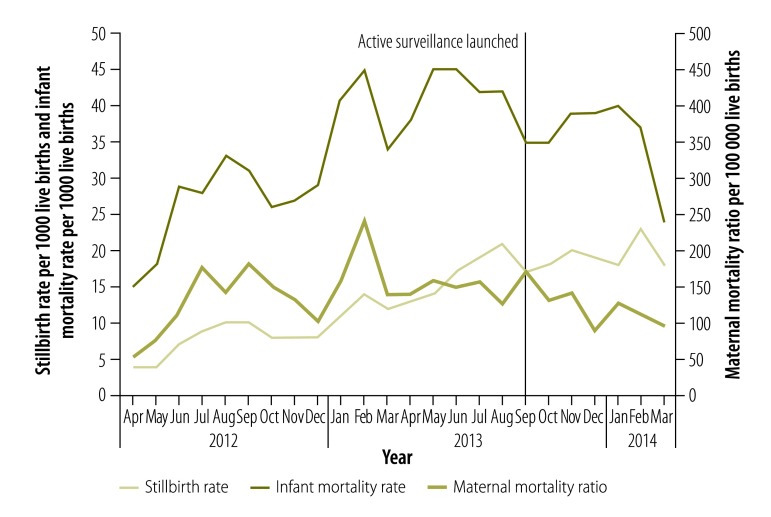
Infant deaths, maternal deaths and stillbirths recorded in Haryana, India, April 2012–March 2014

Notes: All of the data collected before April 2013 came from passive surveillance. In April 2013, the addition of active surveillance was piloted in Karnal district. Subsequently, the combination of passive and active surveillance – i.e. the maternal infant death review system – was gradually rolled out across the state until September 2013, when the whole of Haryana was covered by the system.

Interviews with the relevant officials at state and district levels indicated that the review system had been effective in providing the village-level data needed to make programmatic decisions. The interviewees also discussed how the review system had facilitated supportive supervision. Fig. 2 shows the stillbirth rate, the infant mortality rate and the maternal mortality ratio between April 2012 and March 2014.Trend analysis indicated an improved reporting of infant and maternal deaths across the state. There has been an increase in the reported numbers of maternal and infant deaths in Haryana since the review system was launched. Although the upward trend may indicate increased mortality or increased reporting or both, only 27.8% (242/869) of the maternal deaths and 32.9% (82/249) of the infant deaths registered on the review system’s platform between September 2013 and March 2014 had been recorded on India’s routine health management information system (V Chayal, Post Graduate Institute of Medical Sciences, Rohtak, India, personal communication, 2015).

### Implications

It appears that Haryana’s surveillance-based review system has at least partially addressed the issue of the underreporting of maternal deaths, infant deaths and stillbirths in the state. In complex environments, it becomes essential to have robust measures to record and report mortality – along with other information – to assist health-care monitoring. In many settings, health information systems and related data-sharing mechanisms need to be improved. Haryana’s surveillance-based review system now rapidly provides detailed and relatively accurate information about the health status of women and infants. Reporting from multiple sources has reduced the likelihood of missed deaths and, presumably, enabled better decision-making. Previous systems for recording mortality, such as the civil registration system, have not been very effective.

In some countries, it is not uncommon for more than two thirds of maternal deaths to go unreported in official records.^14^^–^^16^ Low-cost surveillance by key informants can be used as an effective method to monitor trends in maternal mortality, especially in areas with poor vital registration.^17^ In 2004, Tamil Nadu became the first Indian state to establish a system for the mandatory registration and reporting of all maternal deaths, within 24 hours of death, by public and private health facilities.^18^ Kerala and West Bengal have also launched interventions to improve the reporting of maternal deaths and appear to have seen reductions in maternal mortality over time.^19^^,^^20^ In terms of the maternal mortality ratio, Kerala and Tamil Nadu met the 2015 national target – of 100 maternal deaths per 100 000 live births – and Maharashtra is close to achieving the same target.^21^ Haryana’s review system is an attempt to establish a health information system that is similar to those used in Kerala, Maharashtra and Tamil Nadu but tailored to Haryana’s health milieu.

Underreporting of deaths remains an issue across the globe.^22^^–^^31^ A case study in rural Indonesia demonstrated the usefulness of local maternal and child health registers as sources of information in measuring and reporting perinatal mortality and stillbirths, in combination with local vital registration systems.^32^ Even in an urban area of a developed high-income country, such as the United States of America, the use of computer-assisted active surveillance revealed 14 new maternal deaths, resulting in an 88% increase in the ascertainment of maternal deaths.^33^ Other innovative strategies employed to improve the reporting of deaths include record linkage, a retrospective survey among doctors, use of an interview census and confidential forms, specific inquiries on maternal deaths, a household census of birth and infant deaths, matching of hospital records with death certificates and a retrospective population-based survey.^15^^,^^16^^,^^31^^,^^34^^–^^45^

Maternal death review, as implemented in India and several other countries, has led to local policy changes and improvements in the quality of maternal health services, even in challenging settings. Building on this success, WHO introduced maternal death surveillance and response – a continuous action cycle that links routine identification, notification, quantification and determination of causes with actions to prevent future deaths.^46^ While notification of maternal deaths has been mandated by national policy in over 50 countries, only 17 countries have a national mandate for perinatal death reviews.^47^ Although Haryana’s review system has helped improve the quality of the death reports using frontline health workers, there is scope for further improvement in the recording, reporting and notification of deaths. One suggestion is that guidelines similar to those for WHO’s maternal death surveillance and response are followed as a state-level or national policy. One benefit of Haryana’s review system was the creation of a uniform definition of stillbirth across all health worker groups – and this clear definition may well have contributed to the increase seen in the number of stillbirths reported (Fig. 2).

The potential importance of strengthening death – and health – reporting systems, to improve the availability, completeness and quality of the data cannot be overemphasized. Close interaction of health staff with their local communities provides a good foundation for the improvement of death reporting,^48^ especially when passive and active forms of surveillance run in parallel. In Haryana, it remains unclear if the review system will maintain its efficiency if and when the surveillance field volunteers are withdrawn. Refresher training and regular monitoring of the volunteers may be needed to improve their effectiveness. In many places, the frequency of the volunteers’ field visits has gradually fallen. Many of the frontline field workers involved in the review system appear to have very heavy workloads and little time for the detailed documentation of deaths. This problem may have been exacerbated by a lack of awareness about the importance of accurate reporting and inconsistencies in community access to local health-care services.^32^ Further health reforms and policy reviews may well be needed, at both district and state levels, if good data on infant and maternal deaths are to be collected in the long term. An external evaluation of the cost–effectiveness of the review system is still needed.

In general, routine civil registration systems lack political priority and this often leads to inadequate associated policies, poor management and underfunding. Although more effective systems of death reporting may be more complex and require institutional agreements across many governmental departments, they can be made to work given strong regional momentum and leadership.^47^^,^^49^ Given the encouraging results already achieved with minimal external support, Haryana’s successful integrated review system should probably be extended to other states. 
